# Involvement of Adult Hippocampal Neurogenesis in Learning and Forgetting

**DOI:** 10.1155/2015/717958

**Published:** 2015-08-25

**Authors:** Suk-yu Yau, Ang Li, Kwok-Fai So

**Affiliations:** ^1^State Key Laboratory of Brain and Cognitive Sciences, Li Ka Shing Faculty of Medicine, The University of Hong Kong, Hong Kong; ^2^Division of Medical Sciences, University of Victoria, Victoria, BC, Canada V8P 5C2; ^3^Guangdong-Hong Kong-Macau Institute of CNS Regeneration, Jinan University, Guangzhou 510632, China; ^4^Guangdong Key Laboratory of Brain Function and Diseases, Jinan University, Guangzhou 510632, China; ^5^Department of Medicine, Li Ka Shing Faculty of Medicine, The University of Hong Kong, Hong Kong; ^6^Department of Ophthalmology, Li Ka Shing Faculty of Medicine, The University of Hong Kong, Hong Kong

## Abstract

Adult hippocampal neurogenesis is a process involving the continuous generation of newborn neurons in the hippocampus of adult animals. Mounting evidence has suggested that hippocampal neurogenesis contributes to some forms of hippocampus-dependent learning and memory; however, the detailed mechanism concerning how this small number of newborn neurons could affect learning and memory remains unclear. In this review, we discuss the relationship between adult-born neurons and learning and memory, with a highlight on recently discovered potential roles of neurogenesis in pattern separation and forgetting.

## 1. Introduction

The pioneering work demonstrating the continuous generation of adult-born neurons in the hippocampus by Altman in the 1960s [1, 2] has led to extensive investigations on its functions of hippocampus-dependent learning and memory formation in the past decade. Accumulating evidence has implied that adult hippocampal neurogenesis is highly related to some forms of hippocampus-dependent learning and memory of new information [3–5], in addition to its critical role in regulating the hypothalamic-pituitary-adrenal (HPA) axis in response to stress [6], as well as mediating antidepressive effects of antidepressants [7] and physical exercise [8, 9].

Increased adult neurogenesis in the dentate gyrus (DG) of the hippocampus has been shown to improve in memory acquisition, memory formation [10–14], and maintenance [15, 16]. Emerging explorations have focused on clarifying how this small number of newborn neurons can exert a significant impact on the global hippocampal functions and hence learning and memory formation. The functional role of adult-born DG neurons in pattern separation has been increasingly recognized as the key mechanism underlying their influence on learning and memory processing in the hippocampus [17–19]. Pattern separation refers to the processing of similar neural inputs into more distinct and nonoverlapping outputs such that memories, when similar to one another, could be stored without memory interference [20, 21]. While mounting evidence has proven the important role of neurogenesis in pattern separation [17, 22–24], its functional role of adult hippocampal neurogenesis in the clearance of old memories [25–27] is now considered as another mechanism underlying how adult neurogenesis could influence the process of learning and memory in the hippocampus.

Many neurodegenerative and neurodevelopmental disorders involving cognitive impairments are possibly linked to hippocampal dysfunction, which is, at least, partly attributed to dysregulated adult neurogenesis [9]. Therefore, understanding the regulatory processes and the underlying mechanisms of adult neurogenesis in learning and memory formation is of paramount importance for the development of novel clinical cognitive enhancers.

Here we discuss the relationship between adult neurogenesis and hippocampus-dependent learning and memory. We also discuss the recent progress showing how increased neurogenesis improves learning and memory through its essential role in forming pattern separation among similar events, as well as its newly identified role in forgetting past memories.

## 2. The Hippocampus in Learning and Memory

**Figure 1:**
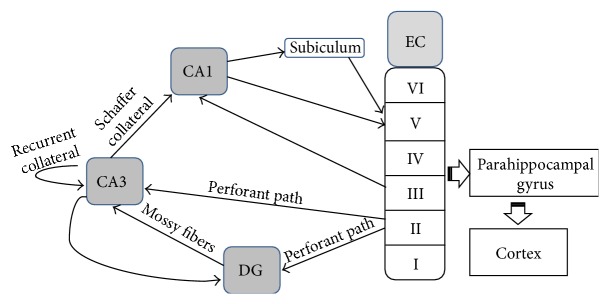
Anatomy of hippocampal network. The diagram illustrates the monosynaptic and the trisynaptic pathways in the hippocampus. The monosynaptic pathway consists of a direct projection from the EC to CA1 or CA3, whereas the trisynaptic pathway consists of sequential projections from EC to DG, CA3, and then to CA1. EC: entorhinal cortex; DG: dentate gyrus; CA: cornu ammonis.

The hippocampus, named for its structural resemblance to a seahorse, is a crucial component of the limbic system and is suggested to be indispensable for various functions, particularly memory acquisition and consolidation and spatial navigation [28, 29]. The hippocampus is composed of four morphologically different subregions, including the DG, Cornu Ammonis (CA), presubiculum, and subiculum [30]. According to the morphological size and appearance of glutamatergic principal cells, which are one of the key cellular types of hippocampal circuits, the CA can be further divided into two major regions: CA1 and CA3 [31]. There are two classical synaptic circuit systems within the hippocampus, namely, the trisynaptic and monosynaptic circuits (Figure 1). The former system is prominently made up of the DG and the CA subregions. Dense axons originating in layers II and III of the EC form the perforant pathway, which forms glutamatergic synapses on dendrites of granule cells in the DG. Thereon, the axons of DG granule cells form the Mossy fiber tract, projecting to the CA3 pyramidal cells whose axons again constitute the Schaffer collateral pathway that ultimately projects back to the subiculum and the EC. Following this principal loop, the sensory input initially received from other parts of the brain is processed and consolidated by the hippocampus and returned to the EC to affect the activity of the whole brain. In terms of the monosynaptic circuit, sparse axons from the EC directly project to the CA1 or CA3 subregion [31].

The hippocampus plays a crucial role in converting short-term memories to long-term memories [32, 33] by processing new memories and temporarily storing them prior to permanent storage in the cortex. Lesion studies have suggested that the hippocampus is important for this temporary storage and the retrieval of contextual fear memory for up to 2-3 weeks after learning [34]. The DG is the first target of the trisynaptic hippocampal circuits. Each dentate granule cell is estimated to make contact with about 12 CA3 pyramidal neurons, which further communicate with approximately 40–60 neighboring pyramidal neurons and 20–30 adjacent inhibitory cells [35–37]. This serves as a perfect amplification of neuronal response within the hippocampus. The DG-CA3 pathway has long been known to be involved in acquisition and consolidation of spatial memory in the Morris water maze (MWM) task [38, 39]. Animal studies with lesions in the DG have demonstrated the crucial role of this subregion in associative memory formation [40, 41] and in the discrimination of similar patterns [42].

## 3. Adult Hippocampal Neurogenesis

Adult neurogenesis in adult mammals occurs in the subgranular zone (SGZ) of the DG [1, 43, 44]. The neural precursors located in the SGZ of the DG are certified to be the origin of newborn neurons [45]. About 1400 newborn neurons are estimated to be added to the bilateral hippocampi of human adults daily, accounting for about 1.8% of the total renewable neuronal population [46]. Likewise, in the young adult rats, approximately 9000 new cells are generated daily, making up to six percent of the total monthly granule cell population [44].

Using retroviral labeling in combination with rabies virus, Vivar et al. indicated that newborn neurons received synaptic input from mature granule neurons with primary innervation from the lateral entorhinal cortex which is responsible for the processing of cued and contextual information [47, 48]. Newborn neurons in this region are able to form synaptic connections with CA3 pyramidal neurons, even if they are not yet fully mature [49]. With their distinct structural [49–51] and synaptic plasticity [52, 53], newborn neurons are poised to play a critical role in forming new memories. The time courses of maturation and functional integration of these neurons are critical for their influences on hippocampal circuits. The maturation of newborn neurons occurs at a faster rate in rats than in mice [54]. During the first week of neuronal maturation, newborn neurons show no synaptic activity in mice [55]. Likewise, in the second week, they display small amplitudes in their action potentials. At the same time, dendritic extensions to the granule cell layer become visible, although no spines have been formed [47, 48, 55]. By the third week, newborn neurons exhibit more physiological properties similar to those of mature cells and now display spine formation [56] and receive excitatory inputs from the mature cells, hilar Mossy cells, and CA3 pyramidal neurons in mice [47]. Newborn neurons in 3-4-week-old mice possess functional properties such as spontaneous action potentials [57], enhanced long-term potentiation (LTP, a cellular form of learning and memory) [58], and synaptic connections to their target pyramidal neurons in the CA3 region [59]. During these developmental stages, newborn neurons in rodents are physiologically distinct from mature neurons whereby they show higher input resistance, reduced GABAergic inhibition, lower induction threshold for LTP, and increased intrinsic excitability [52, 53, 55, 58, 60]. Full maturation of newborn neurons requires several months with continuous growth of spines, dendritic branching, and synaptic connections to target cells.

In addition to synaptic inputs from the lateral and caudomedial parts of the EC, and the perirhinal cortex, newborn neurons also receive back-projections from CA3 pyramidal neurons [48]. Such synaptic connections of newborn neurons to existing circuits within the hippocampus suggest a potential role in the process of memory formation. Myers and Scharfman have proposed that this back-projection may exert inhibitory effects on certain population of DG granule cells; therefore, only a specific population of granule neurons is activated. This may facilitate the sparse coding in the DG when responding to similar patterns and consequently improve pattern separation and memory storage capacity [61]. Of note, the DG-CA3 feedforward inhibition correlates with the quality of memory encoding, given that the increase in this feedforward inhibition plays a critical role in the precision of memory retrieval in contextual fear-conditioning and the MWM tasks, whereas eliminating such inhibition causes memory imprecision [62]. The functional significance of the “back-projection” of the CA3 to the newborn neurons in facilitating learning and memory warrants future investigation.

## 4. Adult Neurogenesis in Hippocampal-Dependent Learning and Memory

Although the estimated daily quantity of integrated adult-born neurons is only ~9000 of the total granule cell population in young rats [44] and ~700 in the unilateral human hippocampus [63], animal studies have shown that this small newborn subset may be sufficient to influence global brain function, based on their ability to encode information, as well as the ability to affect the behaviors of mature neurons by synchronizing the firing and oscillation of neural circuits [43]. The following innate characteristics demonstrate the potential for these newborn neurons to modify the hippocampal circuit. First, newborn neurons are preferentially activated by stimuli, such as spatial learning in the MWM task, as evidenced by the distinct expression profile of immediate early genes in these newborn cells when compared with mature hippocampal neurons [64, 65]. This may be due to the fact that immature newborn neurons display a lower threshold for the LTP induction in response to a theta-burst stimulation [52]. Also, immature adult-born neurons in the mouse at the fourth week of development have a higher probability to be activated by entorhinal inputs, as well as a lower threshold for spiking compared with that of mature granule cells [66]. Second, there is an efficient magnification of active inhibitory synaptic input from adult-born neurons to the local neuronal circuit via a large Mossy fiber connection to CA3 pyramidal neurons, where each newborn neuron can make contact with 11–15 pyramidal neurons via Mossy fibers [67]. Furthermore, one newborn neuron is capable of innervating tens of basket interneurons, which subsequently inhibit hundreds of mature granule cells in the DG [68]. The innervation of Mossy cells in the hippocampal hilus allows a newborn granule cell to contralaterally stimulate many mature granule cells [69]. Third, newborn granule cells can effectively compete for synaptic contact with its target cells and preferentially form synapses with existing boutons. At the age of 30 days, approximately 60% of new cells can form synapses [70]. As a result of this evidence, a small population of newborn neurons may affect global hippocampal functioning, thus having profound effects on learning and memory processes.

### 4.1. Correlation between Hippocampal Neurogenesis and Learning and Memory

As previously discussed, the hippocampus has been considered actively involved in memory formation [71] as well as spatial navigation [29]. Soon after the discovery of adult neurogenesis, Altman hypothesized that this newborn neuronal population in the hippocampal DG might be crucial for learning and memory functions [2]. The initial strategy adopted to reveal the potential relationship between these two events was the analyses on their coincidence, either by manipulating adult neurogenesis to detect the behavioral changes or by conducting behavioral tasks to evaluate changes in neurogenesis.

Levels of neurogenesis in the DG can be modulated by various factors, among which the most frequently employed strategies include environment enrichment [72] and physical exercise as positive regulators [73] and environmental stressors such as chronic unpredictable stress as potent negative regulators [74]. Most evidence has favored the idea that promotion or suppression of hippocampal neurogenesis could correspond with improvement or impairment in learning and memory performances, respectively. For example, 3-month-old Sprague-Dawley (SD) rats housed in an enriched environment for 4–8 weeks showed enhanced hippocampal neurogenesis, which was paralleled by better spatial learning performance in the MWM task [75]. Similarly, adult SD rats that received the identical treatment for a shorter period (17 days) also displayed increased memory in the novel objective recognition test at 24 and 48 hours days after the treatment ended [76]. Similar findings have also been reported in mice. For instance, adult male C57Bl/6 mice housed in an enriched environment for 8 weeks showed promoted hippocampal neurogenesis concurrent with better performance in learning and short-term memory in the MWM task, as well as a higher inhibition in an acoustic startle reflex test [77]. Three-month-old female C57Bl/6 mice subjected to voluntary wheel running for 43–49 days showed increased neurogenesis in the DG; meanwhile, their learning performance in the MWM task and the LTP in the CA1 were both improved [78]. Creer and colleagues reported that 3-month-old male C57Bl/6 mice subjected to running for 38 days showed a specific enhancement of DG-regulated pattern separation in a spatial discrimination test. Notably, the running-induced increase in hippocampal neurogenesis and the improvements in spatial pattern separation were positively correlated with one another [19]. Hippocampal neurogenesis can be modulated through other manipulations, including systemic administration of pharmacological agents such as the herbal drug ginseng [79], the peptide P21 [80], and the neurotrophic factor vascular endothelial growth factor [81], as well as deletion of the Toll-like receptor 3 [82]. Increases in hippocampal neurogenesis in these animals are consistently associated with improvements in their learning and memory performance, in tasks including the contextual fear conditioning, associative passive avoidance, and the MWM [79–82].

In contrast to the positive regulators mentioned above, a variety of negative regulators have been found to impair learning and memory performances. As an example, surgical lesion of the cholinergic septohippocampal pathway reduced hippocampal neurogenesis and significantly compromised spatial learning in a reference memory paradigm [83]. Similarly,* in utero* lipopolysaccharide induction, interruption of rearing, exposure to lead, or insufficiency of vitamin A have all been documented to reduce hippocampal neurogenesis. Such decreases in neurogenesis were coincident with deficits in novel objection recognition, reference memory performance, and formation of fear memories [84–87]. Genetic intervention that retards neurogenesis, such as the deletion of neurotrophin-3 [88], could subsequently cause defects in acquisition, memory retention, and reversal in a reference memory task; notably, it has been suggested that newborn neurons are particularly inclined to be recruited during the process of acquisition and memory retrieval [64]. Hyperactivity of the HPA axis has been known to be involved in the onset of age-related disorders [89, 90]. It has been reported that the magnitude of HPA axis activity in aged animals parallels the level of hippocampal neurogenesis and the reference memory performance [91]. Exposing male rats to prenatal stress that stimulated the HPA axis activity led to the reduction of neurogenesis accompanied by impaired reference memory in the MWM task [92], all of which were reversed by frequent handling in the early postnatal period [93]. Moreover, lowering circulating corticosterone levels by adrenalectomy had an opposite effect on aged rats [94]. Spatial exploration could predominantly activate adult-born neurons compared with their mature counterparts [65, 95], which implies that these newborn neurons may preferentially be involved in information processing [96, 97].

### 4.2. Effects of Blocking Hippocampal Neurogenesis on Learning and Memory Performance

In order to tender more compelling evidence showing the important role of neurogenesis in learning and memory, three different strategies with the same aim of specifically eliminating neural progenitor cells have been employed, including application of antimitotic agents, X-ray irradiation, and genetic manipulations. Following the ablation of neurogenesis, behavioral assays such as the MWM, eight-arm Radial Maze (RAM), and Barnes Maze tasks, as well as the working memory test using the delayed matching to sample (DMS) or delayed nonmatching to sample (DNMS) protocol have been conducted to measure subsequent learning and memory performance. Given that the intervals required for a newborn neuron to fully integrate into existing neural circuits are distinct in mice [55, 57, 59, 98] and rats [54], different protocols have been adopted in the following studies.

Shors et al. provided the first evidence showing that inhibiting neurogenesis by the antimitotic agent methylazoxymethanol (MAM) leads to the impairment of trace, but not delay eyeblink conditioning or contextual fear conditioning [12, 99]. Three weeks after the cessation of MAM administration, such deficits were restored; this suggests that the birth of immature neurons is necessary and also sufficient for trace memory acquisition [99]. Unlike acquisition of spatial reference memory that remained unaltered by MAM treatment [12], retrieval of remote spatial memories in the same MWM task was impaired by blockade of neurogenesis [100]. Other antimitotic drugs, such as 5-fluorouracil [101], cyclophosphamide [102], and temozolomide [103], have been found to exert comparable effects on inhibiting neurogenesis and eliciting behavioral phenotypes in hippocampus-dependent tasks, including the passive avoidance test for fear memory retention, the object location recognition test for spatial working memory, and the MWM.

More recently, X-ray irradiation has been introduced [104] and has been utilized more frequently to ablate neurogenesis due to the greater specificity than that antimitotic agents offer [105]. Forebrain irradiation in adult animals led to the disruption of working memory in the MWM [13] and the RAM tasks [106]. Ko et al. also reported that severe irradiation in adult C57Bl/6 mice compromised short-term memory in contextual fear conditioning [107]. Similarly, Long Evans rats that received irradiation were found to suffer from impairments in long-term memory in the MWM task [16], as well as in short-term memory in contextual fear conditioning [13]. Using the Barnes Maze test to examine spatial reference memory, irradiation has been documented to compromise animal performance [108]. Fan et al. found that learning in the MWM task was impaired following irradiation administration in gerbils [109], similar to another study which examined delayed matching to place behaviors [11].

In the past decade, genetic techniques have been adopted to generate transgenic mice with more restricted targeting to neural progenitor cells. Garcia and colleagues established an inducible glial fibrillary acidic protein- (GFAP-) thymidine kinase (TK) strain where administration of ganciclovir could eliminate neurogenesis [110]. Experiments with these mice revealed that ablating adult hippocampal neurogenesis caused an improved working memory in the RAM task [11, 25] as well as impaired contextual fear conditioning [110]. Additional transgenic models were established with the same target population of nestin-expressing neural progenitor cells. Dupret et al. generated a mouse line with the selective disruption of neurogenesis in the adult hippocampus by doxycycline-inducible overexpression of the proapoptotic protein Bax in nestin-positive neural precursors and detected a compromised acquisition of spatial reference memory in the MWM task [10]. Interestingly, performance relating the cue guidance and egocentric orientation was unaffected, suggesting that newborn neurons in the adult hippocampal DG are needed to build a positional relationship between cues for animals to navigate their environment. Furthermore, novel objective recognition was unaltered, thereby implicating that adult-born neurons may be dispensable for processing simpler forms of spatial information [10, 111]. Tronel et al. provided evidence that this strain of mice without neurogenesis exhibited normal formation and retrieval of associative memory but were unable to discriminate between highly related contexts following the extensive training, suggesting that newborn neurons in the adult hippocampus increase the ability to distinguish highly similar events [112]. The NSE-DTA/Nes-CreER^T2^ transgenic mice, whose neuronal progenitors in the neurogenic regions are eliminated by diphtheria toxin after tamoxifen administration, showed deficits in reference memory retention in the Barnes Maze test and impaired contextual fear conditioning [15]. Deng et al. established another nestin-thymidine kinase mouse line where application of ganciclovir eliminates the dividing neural progenitors [113]. This reduction led to defects in extinction of spatial preference and conditioned contextual fear, as well as long-term retention rather than acquisition of spatial memory [113].

Notably, conclusions obtained by different groups sometimes differ. For example, compromised contextual fear conditioning following suppression of adult hippocampal neurogenesis is reported by some [11, 13, 114, 115], but not by other investigators [10, 12, 116]. Likewise, spatial learning and memory are suggested to be disrupted by some [10, 116], but not by other groups [11, 16, 115]. These discrepancies are potentially owing to the differences in animal species, genetic backgrounds, and behavioral tests, as well as the duration, intensity, and efficiency of methods employed [117, 118]. Thus, advances in research tools with greater specificity, higher efficiency, and more controllable durations of ablation are preferred for future in-depth mechanistic studies [118].

## 5. Hippocampal Neurogenesis Improves Pattern Separation 

As a gateway for information's entry to the hippocampus where memories are retained in associative networks [119], the DG has been indicated as the core structure enabling pattern separation [120]. Pattern separation in the DG occurs when highly similar input firing patterns are processed into less similar output firing patterns within the network. This can happen with either different firing rates within a population of granule cells or firing of different subpopulations of granule cells in response to a network input. The DG comprises at least five- to tenfold more neurons compared with the EC, which allows information to be projected into higher-order structures, and thus enhances learning discrimination [121]. In addition, the dentate granule cells are often inactive during behavioral tests [122, 123], possibly due to feedback inhibition of the neural circuit accomplished by local interneurons [124]. This sparse coding pattern enables the DG to separate the overlapping inputs and produce a spare representation from fewer neurons in response to resembling inputs [20, 125]. Moreover, activation of individual granule cells in the DG, although sparse, is capable of relaying information by depolarizing pyramidal cells in the CA3 region through Mossy fibers [126], subsequently facilitating memory encoding [119].

With the aforementioned features of the DG, the pattern-separation function of adult-born hippocampal neurons has emerged as the neurobiological basis mediating the influence of adult hippocampal neurogenesis on learning and memory [18, 127]. Nakashiba et al. have reported that older granule neurons are required for discriminating relatively distinct contexts, whereas young neurons are required for the fine discrimination of similar contexts in mice [22]. They have suggested a functional shift from pattern separation to pattern completion as neurons age [22]. From this point of view, continuous adult neurogenesis is needed for distinguishing similar events and avoiding memory interference when new memories are formed. In agreement with this opinion, recent studies have shown that abrogating neurogenesis by irradiation impairs the performance of mice in a space separation behavioral task involving distinguishing contiguous but not far-separated targets [17, 112]. Likewise, chemically ablating hippocampal neurogenesis by temozolomide leads to poor performance of mice in the MWM task, showing difficulties in memorizing new positions of the hidden platform, concurrent with prolonged retention of the old memory, as reflected by the greater inclination to swim to the old platform position [103]. Conversely, increasing adult neurogenesis by knocking out the proapoptotic gene Bax in neural progenitor cells improves the discrimination of representations that contextually overlap [18]. Similar results have also been observed in adult mice following exercise, which display an enhancement of neurogenesis [19]. Increase in neurogenesis and improvements in learning and memory elicited by physical exercise [128] are paralleled by increased production of brain-derived neurotrophic factor (BDNF) [129]. Bekinschtein et al. have reported that BDNF in the hippocampal DG plays a critical role during encoding of pattern-separated memories [130]. They have recently shown that direct infusion of BDNF into the DG increased spatial discrimination in control rats, whereas blockade of neurogenesis diminished these improvements [131], suggesting that adult-born neurons are required for BDNF-enhanced pattern separation [24].

Computational models have proposed that pattern separation processing within the hippocampus involves the DG-CA3 circuit [132]. Through the use of chemical or genetic approaches to ablate hippocampal neurogenesis, different contexts are suggested to be coded by distinct subpopulations of CA3 neurons [133, 134]. Niibori et al. have shown that the absence of hippocampal neurogenesis caused behavioral impairment in contextual discrimination [23]; they have also demonstrated that suppression of adult neurogenesis impaired the population coding of similar contexts in the CA3 region. These data suggest that adult neurogenesis may facilitate population coding in the CA3, thus enhancing the process of pattern separation in the hippocampus. As previously discussed, the DG-CA3 feedforward inhibition correlates with the accuracy of memory encoding [62]; it is therefore reasonable to speculate that the back-projections from CA3 to immature neurons may play a critical role in facilitating the sparse coding by DG granule cells [61].

A recent human study has reported that following six weeks of physical exercise training, individuals performed better in a hippocampus-dependent visual pattern-separation task, and a lower depression score was recorded compared with those who did not exercise [135]. On the other hand, the age-related cognitive decline may be partially attributed to the decrease in hippocampal neurogenesis, given that both animals and human subjects have been confirmed to experience a significant suppression of adult hippocampal neurogenesis with ageing [136, 137]. Stark et al. have reported that healthy human subjects displayed an age-related decline in pattern separation, but not in recognitive memory performance, whereas those diagnosed with mild cognitive impairment showed reductions in both scores [138]. Since changes in the DG have been found in aged human and rodent brains [139], it is likely that ageing-induced decreases in neurogenesis may partly contribute to the impaired function of pattern separation in the senile population. Holden and colleagues reported a decreased efficiency in spatial pattern separation in older adults in comparison to young adults, which could be due to age-related changes in the DG and CA3 regions [140].

## 6. Hippocampal Neurogenesis Improves the Forgetting of Old Memories

Mounting evidence over the past decade has shown that alterations in adult neurogenesis are a form of plasticity that improves hippocampus-dependent learning and memory formation. Of note, a recent study has unveiled a new role for adult neurogenesis in promoting forgetting of old memories [141]. In memory processes, animals may need to unlearn or inhibit the learned task by modifying the existing memory trace, such that new memories can be learned and stored. Emerging trends in the functional role of neurogenesis on learning and memory have suggested that production of newborn neurons may modulate the hippocampal network to form and store new memories, which may require the clearance of old memories in order to optimize the capacity for learning and memory processes.

Feng et al. 2001 have shown that environmental enrichment prior to learning increases hippocampal neurogenesis and improves performance in both contextual and cued fear-conditioning tests in forebrain-specific presenilin-1 knockout (PS1-KO) mice with impaired neurogenesis [142]. However, the introduction of environmental enrichment for 2 weeks after the acquisition phase increased freezing responses in the contextual retention test in the PS1-KO, but not in the wild-type littermates, suggesting that eliminating neurogenesis facilitates the retention of contextual fear memories. The authors postulated that deficits in hippocampal neurogenesis may prevent the clearance of contextual memory traces, which consequently resulted in improved memory retrieval. Coincidentally, another study conducted by van der Borght et al. showed that enhanced hippocampal neurogenesis following physical wheel running prior to a learning task in the Y-maze was associated with improvements in acquisition, retention, and reversal learning [143]. Such a finding was in favor of the positive correlation between learning and neurogenesis, as reported by Feng et al. [142]. However, when reapplying running exercise to mice after the initial training session, van der Borght et al. reported improvements in both memory retention and retrieval [143]. The discrepancy between these two studies may be due to different learning tasks (Y-maze versus contextual fear conditioning) and distinct strategies to stimulate neurogenesis (voluntary running versus environmental enrichment). Of note, van der Borght et al. showed that memory retention and reversal learning significantly reduced neurogenesis in both runners and nonrunners [143]. Since memory retention and reversal learning require recall of the information previously acquired, the authors hypothesized that such decrease in neurogenesis may help prevent interference between previously and newly formed memories. Hence, inhibiting neurogenesis potentially assists in optimizing memory retrieval. In fact, Saxe and colleagues have reported that too much neurogenesis could actually be harmful to hippocampus-dependent working memory, which is a form of short-term memory involving both the hippocampus and the prefrontal cortex [25]. Following ablation of hippocampal neurogenesis by either low-dose X-irradiation or a genetic approach (e.g., ganciclovir-induced elimination of neurogenesis in the GFAP-TK mice), an improvement of working memory was detected in the RAM task that examined the ability to discriminate highly similar cues with an intertrial delay longer than 30 sec [25].

Evidence supporting both theories of neurogenesis contributing to the clearance of old memories or the formation of new memories has been reported. For example, in a previous study exploring how neurogenesis modulates hippocampal network activity to enable memory storage at different levels, Deisseroth and colleagues demonstrated an activity-sensing property of hippocampal neural progenitor cells via Cav1.2/1.3 (L-type) Ca^2+^ channels and NMDA receptors, suggesting that excitation of the local neural network may regulate the neurogenic process [27]. In fact, such activity-dependent responses during hippocampal neuronal generation potentially contribute to the formation of new memories and the clearance of old memories [27]. Their study also showed that clearance of old memories could be accelerated by enhancing neurogenesis, whereas neurogenesis is needed for the formation of new memories, especially in a situation of higher hippocampal network activity [27]. Together, they tendered evidence supporting the idea that appropriate levels of neurogenesis, which coincides with the excitatory network activity, may be advantageous for the hippocampus to balance old memory storage and new memory formation. Given that the hippocampus actively participates in the formation of new memories and temporary memory storage, a timely elimination of old memories will improve the efficiency in forming and storing new memories with the existing hippocampal network [27]. Based on this theory, it is anticipated that overstimulating hippocampal neurogenesis may increase the degradation of old memories and subsequently result in memory deficits. Accordingly, levels of hippocampal neurogenesis should be tightly regulated by network activity. The aforementioned back-projection from CA3 to DG discovered by Vivar et al. may have inhibitory effects on activity of newborn neurons for old memory retrieval [47]. Future research on verifying this assumption is of great interest.

Akers and colleagues tested if the performance in memory retention could correlate the level of hippocampal neurogenesis [141], by comparing behavioral performances in memory retention between adult mice and pups, whose neurogenesis is at low (in adulthood) and fairly high levels (in the early postnatal period, around 17 days old), respectively. In that study, mice were subjected to fear-conditioning training where they received foot shocks in a novel context, followed by assessments in the same context from Day 1 up to 6 weeks without foot shocks. By measuring the freezing duration in the same context, the adult mice showed the stable memory retention throughout the test period, whereas pups first exhibited the comparable memory retention on Day 1 but experienced a quick decline after a week. Interestingly, the increase of neurogenesis after fear conditioning by either the voluntary wheel running or treatment with the antidepressant fluoxetine promoted forgetting in adult mice. In contrast, reducing postnatal neurogenesis in infant mice after fear conditioning led to improvements in memory retention. They further reported the correlation between neurogenesis and forgetting, as evidenced by the data that guinea pigs and degus with low levels of postnatal neurogenesis owned the intact memory retention but displayed forgetting when their hippocampal neurogenesis was enhanced by memantine. Collectively, this study indicates that forgetting is impaired in infant rodents with low levels of hippocampal neurogenesis, while increasing neurogenesis can induce forgetting.

Based on the hypothesized role of adult neurogenesis in memory clearance, it is reasonable to speculate that suppression of this process, though impairing the formation of new memories, may facilitate the preservation or reconsolidation of previously formed memories. Kitamura and colleagues have demonstrated that inhibition of neurogenesis by X-ray irradiation prolonged the maintenance of LTP in DG, as well as preservation of old memories (up to 30 days after learning) in the contextual fear-conditioning task; this suggests that the hippocampus-dependent memory retention could be extended by inhibiting neurogenesis [26]. Although this study has tendered evidence supporting the regulatory role of old memory decay by hippocampal neurogenesis, whether this also affects memory consolidation to the cortex remains unclear.

Computational models predict that encoding new information will not only remodel neural networks, but also weaken neural connections that have already been established for storing old memories [144]. Increased neurogenesis with the concomitant loss of old memories may be due to the fact that immature granule cells compete for synaptic contact with mature neurons. By forming synaptic connections preferentially with the existing boutons, adult neurogenesis gets promoted and then intensifies synaptic competition, which leads to fewer synaptic inputs into existing individual neurons. The prior morphological studies have shown that increasing adult neurogenesis does not change the number of synapses but decreases the excitatory transmission to mature granule cells due to fewer synapses formed with mature granule cells [59, 145]. The discussed study by Akers et al. [141] echoes the assumption that functional integration of newborn neurons may result in circuit modifications that compete with the preexisting circuits, contributing to forgetting of existing memories. To encode new memories dependent on adult neurogenesis, as well as to retain old memories already formed, a threshold of adult neurogenesis may allow an optimal performance for both learning and memory formation [146]. Taking all findings together, it is reasonable to predict that adult neurogenesis may function as a key regulator of new memory formation and old memory decay in the hippocampus. Increasing hippocampal neurogenesis may facilitate acquisition by reducing the interference of similar memories (pattern separation) and by concomitantly reducing pattern completion for old memory retrieval (forgetting of old memories), and* vice versa* [147].

## 7. Conclusion

**Figure 2:**
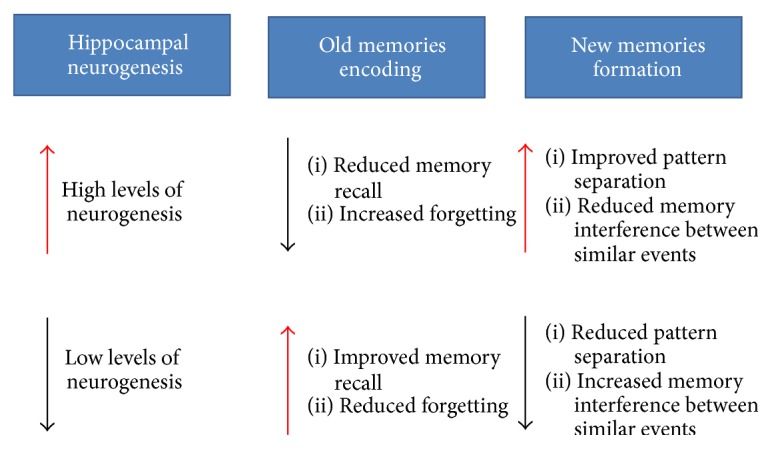
Potential influences of adult neurogenesis on new memory formation and old memory clearance. Increased neurogenesis improves pattern separation when acquiring new information with much overlap and yet accelerates clearance of old memories. Conversely, decreased neurogenesis facilitates the temporal storage of short-term memory and thus enhances memory retrieval in the hippocampus, yet aggravating memory interference of similar events during new information acquisition.

Based on the evidence from both animal and human studies, investigations on adult neurogenesis have been answering the critical questions concerning how learning and memory are formed and regulated in adult mammalian brains. Pattern separation and forgetting induced by adult neurogenesis may be the way in which the brain normally learns and retrieves memory. A computational model used by Weisz and Argibay [148] demonstrated that learning itself increases the number of granule cells, whereas the retrieval of recent memories can still be improved with blockade of hippocampal neurogenesis; this suggests that neurogenesis can promote the hippocampal network capacity for new information and enhance the clearance of old memories. They later hypothesized that the addition of hippocampal adult-born neurons contributes not only to the successful neural adaptation to the environment with pattern separation and pattern integration for forming new memories, but also to the interference while retrieving old memories [149]. Emerging evidence from both theoretical and experimental studies has suggested the influences of adult neurogenesis on pattern separation for learning new information, as well as on interference with old memory retrieval that results in forgetting. Adult neurogenesis in the hippocampus may serve as a normal cellular process for learning and memory consolidation. Excessive addition or insufficient generation of newborn neurons may lead to abnormal clearance of old memories or failure in forming new memories in the hippocampus, respectively, subsequently disrupting memory process and storage in the brain (Figure 2). Therefore, changes of neurogenesis, either excessive or inadequate, may be deleterious to learning and memory. This raises the possibility that only when a threshold of adult neurogenesis is reached will the acquisition of new information be facilitated. Revealing how much increase or decrease of neurogenesis is appropriate for a good trade-off between new and old memories is of great interest for future research.
